# Reorganization of cortical oscillatory dynamics underlying disinhibition in frontotemporal dementia

**DOI:** 10.1093/brain/awy176

**Published:** 2018-07-09

**Authors:** Laura E Hughes, Timothy Rittman, Trevor W Robbins, James B Rowe

**Affiliations:** 1 Department of Clinical Neurosciences, University of Cambridge, UK; 2 Medical Research Council Cognition and Brain Sciences Unit, University of Cambridge, UK; 3 Department of Psychology, University of Cambridge, Cambridge, UK; 4 Behavioural and Clinical Neuroscience Institute, Cambridge, UK

**Keywords:** frontotemporal dementia, magnetoencephalography, response inhibition, cross-frequency coupling, beta desynchronization

## Abstract

The distribution of pathology in frontotemporal dementia is anatomically selective, to distinct cortical regions and with differential neurodegeneration across the cortical layers. The cytoarchitecture and connectivity of cortical laminae preferentially supports frequency-specific oscillations and hierarchical information transfer between brain regions. We therefore predicted that in frontotemporal dementia, core functional deficits such as disinhibition would be associated with differences in the frequency spectrum and altered cross-frequency coupling between frontal cortical regions. We examined this hypothesis using a ‘Go-NoGo’ response inhibition paradigm with 18 patients with behavioural variant frontotemporal dementia and 20 healthy aged-matched controls during magnetoencephalography. During Go and NoGo trials, beta desynchronization was severely attenuated in patients. Beta power was associated with increased impulsivity, as measured by the Cambridge Behavioural Inventory, a carer-based questionnaire of changes in everyday behaviour. To quantify the changes in cross-frequency coupling in the frontal lobe, we used dynamic causal modelling to test a family of hierarchical casual models, which included the inferior frontal gyrus, pre-supplementary motor area (preSMA) and primary motor cortex. This analysis revealed evidence for cross-frequency coupling in a fully connected network in both groups. However, in the patient group, we identified a significant loss of reciprocal connectivity of the inferior frontal gyrus, particularly for interactions in the gamma band and for theta to alpha coupling. Importantly, although prefrontal coupling was diminished, gamma connectivity between preSMA and motor cortex was enhanced in patients. We propose that the disruption of behavioural control arises from reduced frequency-specific connectivity of the prefrontal cortex, together with a hyper-synchronous reorganization of connectivity among preSMA and motor regions. These results are supported by preclinical evidence of the selectivity of frontotemporal lobar degeneration on oscillatory dynamics, and provide a clinically relevant yet precise neurophysiological signature of behavioural control as a potential pharmacological target for early phase experimental medicines studies.

## Teaser

Impulsivity and disinhibition are major symptoms of frontotemporal dementia, with limited treatment options. Using magnetoencephalography, Hughes *et al.* characterise the neural oscillatory dynamics of response inhibition, revealing impaired beta-band desynchronization and disrupted cross-frequency coupling in patients. This cortical oscillatory signature of disease represents a potential pharmacological target for symptomatic treatment.

**See Meder and Siebner (doi:10.1093/brain/awy195) for a scientific commentary on this article.**

## Introduction

A major challenge to understanding behavioural changes and restoring function in frontotemporal dementia (including behavioural variant frontotemporal dementia, bvFTD) is to establish the mechanistic links between neuropathology, brain function and behaviour. Neural networks are selectively vulnerable to dementias and the network paradigm of cortical circuit reorganization provides a sensitive and specific index of the functional sequelae of neuropathology (Seeley *et al.*, 2009; Rowe, 2010; Zhou *et al.*, 2010; Cope *et al.*, 2018). Here we advance the network paradigm of dementia to incorporate emerging preclinical evidence of specific signatures of oscillatory dynamics.

In health, brain connectivity is hierarchically organized at multiple scales, including the laminar cytoarchitecture: the flow of information between cortical regions is regulated principally by the projections between supragranular and infragranular layers (Barbas, 2015; Jensen *et al.*, 2015). Feedforward and feedback connections originating in superficial and deep layers have distinct spectral fingerprints, preferentially oscillating at high and low frequencies, respectively (Kopell *et al.*, 2010; Buffalo *et al.*, 2011). The interactions between frequency bands (known as cross-frequency coupling) represent integration of information across spatial and temporal scales (Canolty and Knight, 2010) and can reveal the direction of information flow (Hillebrand *et al.*, 2016). The different frequency bands are mediated by complex neurochemistry; however, gamma oscillations are dependent on circuits of GABAergic neuronal inhibition (Bartos *et al.*, 2007; Buzsaki and Wang, 2012). Of particular relevance, the neuropathology in bvFTD is not uniform in the cortex—there is preferential cell loss from supragranular layers (Kersaitis *et al.*, 2004; Irwin *et al.*, 2016), including a reduction in GABAergic neurons (Murley and Rowe, 2018). Sami *et al.* (2018) reveal different pathologies, including Alzheimer's disease and frontotemporal dementia, have a specific ‘signature’ in the oscillatory frequency of communication in brain networks. Measureable changes in frequency-specific bandwidths might provide the key mechanistic link between the neuropathological specificity of bvFTD and impaired behaviour. For example, transgenic models of FTD have revealed shifts in the frequency spectrum related to behaviour (Koss *et al.*, 2016). Electrophysiological measurements have the advantage of providing a temporally precise estimate of oscillatory dynamics in the context of cognitive and behavioural tasks. To identify frequency specific changes in relation to behaviour, we used magnetoencephalography (MEG) during a task of response inhibition.

Disinhibition is a core feature of bvFTD, resulting in impulsive and inappropriate behaviours. It is a criterion for bvFTD (Rascovsky *et al.*, 2011), and is common throughout the spectrum of disorders associated with frontotemporal lobar degeneration (Coyle-Gilchrist *et al.*, 2016; Lansdall *et al.*, 2017). The inappropriateness of many behaviours may arise from the profound deficits in social cognition and personality that typically occur in FTD (Ibanez and Manes, 2012), raising the possibility of a detrimental synergy between failures of behavioural inhibition and social cognition.

Disinhibition within the context of a social and emotional model of FTD arises from dysfunction in a network of orbitofrontal, prefrontal, insular, and temporal cortices, together with amygdala and striatum (Ibanez and Manes, 2012) and underlies many of the social and emotional deficits observed in bvFTD (Santamaria-Garcia *et al.*, 2017). For example, grey matter atrophy and loss of white matter tracts in orbito and medial frontal regions, as well as in the temporal lobe is directly related to performance on neuropsychological tests of disinhibition (Hornberger *et al.*, 2011; Lansdall *et al.*, 2018). However, the mechanisms of how these disinhibited behaviours manifest, via control from affected brain regions to motor circuits, are important in understanding and treat behavioural change.

In health, successful response inhibition invokes a large-scale brain network centred on the right prefrontal gyrus (IFG) (Aron *et al.*, 2014) and presupplementary area (preSMA) (Rae *et al.*, 2015). There are several mechanisms by which the IFG may exert control over behaviour, including behaviours that are socially and emotionally inappropriate. Electrophysiological evidence suggests the importance of frequency-specific reciprocal connectivity between prefrontal, premotor and motor cortex in the beta (12–30 Hz) (Picazio *et al.*, 2014) and gamma ranges (>30 Hz) (Muthukumaraswamy, 2010; Joundi *et al.*, 2012). A characteristic change in beta power particularly associated with movement control is an event-related neural desynchronization (ERD) in the beta bandwidth that precedes movement execution and inhibition, followed by a subsequent increase in beta power (beta rebound or event-related synchronization, ERS) (Pfurtscheller and Lopes da Silva, 1999; Neuper *et al.*, 2006; Solis-Escalante *et al.*, 2012). In several neurological diseases, such as Parkinson’s disease and amyotrophic lateral sclerosis, beta power is significantly altered (Brown and Marsden, 1999; Schnitzler and Gross, 2005; Levy *et al.*, 2010; Bizovicar *et al.*, 2014) suggesting that this frequency represents an essential feature for motor control. Beta oscillations are also noted to be key in feedback interactions between the IFG and motor areas, especially during response inhibition (Picazio *et al.*, 2014). In conjunction with beta oscillations, increases in gamma power are also observed during action control, which may facilitate responses (Muthukumaraswamy, 2010; Joundi *et al.*, 2012).

**Figure 1:**
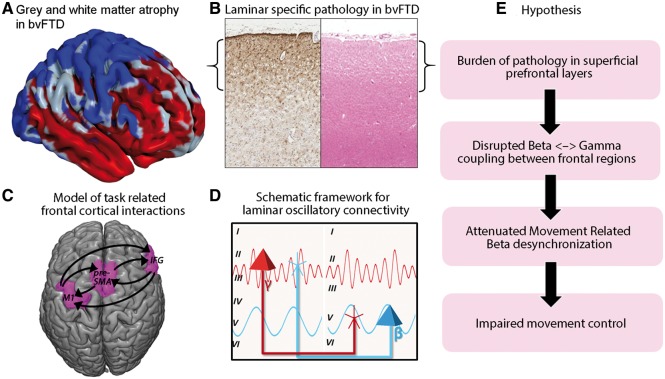
**Illustration of the experimental background and principal hypothesis.** (**A**) Voxel-based morphometry of grey and white matter loss in the patient versus control group. The areas in red confirm the expected reductions in grey and white matter tissue in frontal and temporal cortex. The areas coloured blue had strong evidence for normal cortical volume (Bayesian probability of the null > 0.7). While prefrontal regions are particularly atrophic (red), the precentral gyrus, including the primary motor cortex, had evidence of normal grey matter volume (blue). (**B**) GFAP (*left*) and haematoxylin and eosin (*right*) slices from frontal cortex in a patient with bvFTD, which demonstrate a clear outer layer emphasis to the pathology (as indicated by the brackets). (**C**) A model of the regions included in the DCM analysis (IFG, preSMA and motor cortex), and the specified connectivity. (**D**) Schematic illustration of the framework for the experimental motivation: superficial layers of prefrontal cortex generating gamma oscillations have reciprocal connections with the deeper layers of motor regions which support beta oscillations. (**E**) Hypothesis: the layer-specific burden of pathology is predicted to disrupt the cross-frequency coupling, attenuate the beta desynchronization and consequently impair movement control.

Here we used MEG to examine the impact of bvFTD on frequency-specific changes in relation to behaviour, and connectivity between prefrontal, premotor and motor cortex. We used the Go-NoGo task to assess inhibitory control, based on the extensive evidence of its linkage of clinical tests (Dubois *et al.*, 2000; Torralva *et al.*, 2009), carers’ reports of behaviour (Hughes *et al.*, 2015), systems neuroscience (Aron *et al.*, 2014) and psychopharmacological strategies for therapy (Hughes *et al.*, 2015; Ye *et al.*, 2016). To identify network connectivity and quantify the parameters (including cross-frequency coupling) we used formal measures of evidence from hierarchical generative models of frontal brain networks. We predicted that (i) bvFTD impairs the beta desynchronization and resynchronization pattern, in association with clinically meaningful disinhibition; and (ii) bvFTD alters the beta and concomitant gamma oscillations as a result of changes in cross-frequency coupling between regions of the frontal cortical network for behavioural control. The hypotheses and rationale are summarized in Fig. 1.

## Materials and methods

### Participants

Eighteen right-handed adult patients with bvFTD were recruited from the specialist frontotemporal dementias clinic at the Cambridge University Hospitals NHS Trust. Diagnosis of bvFTD was made by a consultant neurologist in a multidisciplinary clinic, based on the international consensus clinical diagnostic criteria described by Rascovsky *et al.* (2011), including at least three of six core criteria with progressive deterioration of behaviour/cognition reported by a caregiver, functional and neuropsychological impairments, and abnormal structural MRI (Rascovsky *et al.*, 2011). Patients with other types of dementia, or primary language or motor deficits were not included. Patients did not meet criteria for other major psychiatric disorders. The patients were investigated on their usual medication. A control group of 20 right-handed age-matched healthy older adults were recruited from the volunteer panel of the MRC Cognition and Brain Sciences Unit. None had a history of significant neurological or psychiatric illness. The study was approved by the local Research Ethics Committee and all participants gave written informed consent according to the 1991 Declaration of Helsinki.

Participants underwent neuropsychological assessment during or close to the day of MEG, including the revised Addenbrooke’s cognitive examination (ACE-R) (Mioshi *et al.*, 2006), the Mini-Mental State Examination (MMSE), and the Hayling and Brixton Task (Burgess and Shallice, 1997). Caregivers completed the Cambridge Behavioural Inventory (CBI) (Wedderburn *et al.*, 2008) and the abbreviated Neuropsychiatric Inventory (Cummings *et al.*, 1994). Details are summarized in Table 1.

**Table 1:** Details of bvFTD patients and healthy controls

	Controls	bvFTD	Group difference
**Male/female**	8/12	12/6	n.s.
**Age**	61 (9.32)	63 (6.6)	n.s.
**MMSE**	29 (0.9)	23 (5)	*P* < 0.001
**ACE-R**			
Total (100)	97 (2.1)	66 (18.3)	*P* < 0.001
Attention (18)	18 (0.7)	14 (3.4)	*P* < 0.001
Memory (26)	25 (1.1)	15 (7.0)	*P* < 0.001
Verbal fluency (14)	13 (1.2)	4 (2.9)	*P* < 0.001
Language (26)	26 (0.4)	19 (6.5)	*P* < 0.001
Visual spatial (16)	16 (0.8)	13 (2.4)	*P* < 0.001
**CBI**			
Total	–	105 (41.1)	
Disinhibited phenotype scale^a^	–	38 (18.1)	
**Hayling**			
A+B Errors	–	23 (18.8)	
**Graded Naming Test**			
No. correct (total 30)	–	11 (7.8)	
**Neuropsychiatric Inventory****^b^**			
Total symptoms (12)	–	5.5 (2)	
Total severity (36)	–	10.11 (4.6)	
Total distress (60)	–	12.2 (7.0)	

Values shown are group means (SD in parentheses). MMSE = 30-point Mini-Mental State Examination; ACE-R = Addenbrooke’s cognitive exam revised, scored out of 100, divided into five subscales with total points for each in parentheses. The Hayling score is the converted error score on section two ‘unconnected completion’. Group differences were tested using Mann Whitney U-test.

^a^Composite sum from Cambridge Behavioural Inventory (CBI) subscales including all items from the disinhibited, challenging, motor, eating and insight subscales, and the euphoria items from the mood subscale (Borroni *et al.*, 2012; Hughes *et al.*, 2015).

^b^Ten of 18 patients completed the abbreviated version of the Neuropsychiatric Inventory.

### Task

The Go-NoGo task has been described in detail previously (Hughes *et al.*, 2015). Briefly, it comprised 400 Go trials and 104 NoGo trials, visually cued with the symbols ‘O’ or ‘X’, respectively, presented centrally until a response, or until 1.5 s if no response was made. Each trial started with a fixation cross presented centrally on a dark grey background for 2 s. Letter cues subtended 0.8°. Participants were instructed to look at the fixation cross and press a button with their right hand as quickly as they could to the Go cue and to withhold their press to the NoGo cue. Trial order was pseudorandom, permuted such that on 20% of trials a NoGo cue was presented after a series of one to eight Go trials, or immediately after a previous NoGo trial. Presentation of stimuli was controlled using EPrime^®^. Before the MEG recording, all participants were given 40 practice trials, and we confirmed that they had understood the task.

### MEG data collection

MEG data were acquired continuously at 1 kHz in a magnetically-shielded room with a 306-channel Vectorview MEG system (Elekta Neuromag), which contains two orthogonal planar gradiometers and one magnetometer at each of 102 positions. Five head position indicator (HPI) coils were used to monitor head position. Vertical and horizontal electrooculograms were recorded using paired EOG electrodes. The 3D locations of the HPI coils, 80 ‘head points’ across the scalp, and three anatomical fiducials (the nasion and left and right pre-auricular points), were recorded using a 3D digitizer (Fastrak Polhemus Inc.).

The raw MEG data were initially preprocessed using MaxFilter software (version 2.2, Elekta-Neuromag) with movement compensation. Further preprocessing and data analysis used MATLAB (The MathWorks, Natick, MA) and SPM12. Data were downsampled to 500 Hz and eye-blink artefacts were corrected using the Berg method of artefact correction (a topography-based artefact correction method) (Berg and Scherg, 1994), and high-pass filtered above 0.1 Hz. Epochs of 2500 ms were extracted (−500 ms to 2000 ms) time-locked to the stimulus onset. Epochs containing artefacts were rejected if the amplitudes exceeded the following thresholds: 2500 fT for magnetometers and 900 fT for gradiometers. After artefact rejection the mean number of trials included for the accurate Go and NoGo conditions for the control group was 380 [standard error (SE) = 7.7] and 93 (SE = 2.5), respectively; and for the patient group 379 (SE = 20.1) and 92 (SD = 5.0). Forward modelling with dynamic causal modelling (DCM) was estimated using cortical meshes based on co-registering the fiducials and head shape points to the participant’s structural MRI scan.

### Data analyses

#### Behaviour

Behavioural analyses examined mean reaction time for correct Go and incorrect NoGo responses, and response accuracy (arsine transformed) using IBM SPSS Statistics 22.0^®^. Reaction times and accuracy rates are presented in Table 2. Independent two-sample *t*-tests compared reaction times of the patients to controls, and Mann-Whitney U-tests were used to compare response accuracy (due to non-Gaussian distribution). Greenhouse-Geisser correction was used to correct for non-sphericity where necessary. Cohen’s *d* effect size is also reported. We supplement classical frequentist classical statistics with a Bayesian analysis of group differences using JASP software to test the hypothesis that patients are slower in reaction times and less accurate. Thresholds for interpretation are Bayes factors 3, 20 and 150 representing weak, strong and very strong evidence, respectively.

**Table 2:** Mean reaction times and accuracy rates (arcsin transformed in radians, and non-transformed mean accuracy %) for Go (correct trials) and NoGo (commission errors) trials

	**Controls (*n* = 20)**	**bvFTD (*n* = 18)**
**Reaction times (ms)**		
Go	289.7 (9.1)	500.4 (42.9)
NoGo	223.7 (12.7)	445.4 (72.9)
**Accuracy (rad)**		
Go	1.5 (0.01)	1.3 (0.02)
NoGo	1.4 (0.04)	1.3 (0.05)
**Accuracy (%)**		
Go	99.2 (0.4)	92.7 (1.2)
NoGo	93.8 (1.3)	90.5 (3.4)

Standard errors are in parentheses.

An index of clinical behavioural disinhibition was calculated from the CBI, including the sum of all items from the disinhibited, challenging, motor, eating and insight subscales, and the euphoria items from the mood subscale (Hughes *et al.*, 2015). These specific types of behaviours have been previously shown to robustly quantify the syndrome of behavioural ‘disinhibition’ (Borroni *et al.*, 2012). This index was used in correlations with the time-frequency data.

#### Time-frequency in sensor space

Time-frequency power spectra were computed for frequency bands between 4–80 Hz across the whole epoch using Morlet wavelets with a factor of 5. The transformed data were baseline corrected using a log ratio of power and scaled to dB. Only the accurate trials were used in the data analysis, as a measure of the physiological thresholds for movement (Go Trials) and withholding movement (NoGo trials). The number of accurate trials (but not inaccurate trials) was sufficient for good signal-to-noise ratio and power for MEG analyses.

Statistical analysis was performed on 2D images of frequency by time, averaging across the root mean squared value of gradiometer sensor pairs. These images were entered into a 2 × 2 ANOVA, to test the differences and interactions between the conditions and groups. The statistical maps were thresholded with a cluster-based family-wise error (FWE) correction *P* < 0.05 (after *P* < 0.001 voxel-wise height threshold).

#### Network modelling

DCM for MEG is an approach that explains the statistical dependencies between sources in terms of causal mechanisms, by inversion of generative models of brain networks to the neurophysiological observations. For the analysis of induced responses, DCM provides a phenomenological model of the time-dependent changes in spectral density. Importantly, this method captures how the frequency dynamics in one source affect the same or different frequency dynamics in another source, thus revealing both linear (within frequency) and non-linear (cross-frequency) couplings.

A detailed explanation of this method is described by Chen *et al.* (2008, 2009). In summary, DCM involves three main stages of analysis. The first step is the architectural specification of the neuronal network model, in which the neuronal sources of interest are identified and defined by MNI coordinates and the connections between sources varied to create a set of models to compare. The number of sources included in DCM is limited due to computations becoming intractable with large numbers. The second step is the inversion of the model to the observed data, and the time-frequency decomposition. The sources are modelled with equivalent current dipoles and then spectral density is calculated using a Morlet wavelet transform. For computational efficiency data are reduced to a number of modes from a singular value decomposition of the spectral power. The coupling dynamics between regions (i.e. time-dependent changes in spectral energy for each connection) are estimated using linear state equations. These estimates are represented in ‘A’ and ‘B’ matrices for each connection. If the model is specified as only ‘linear’, then each matrix will represent within frequency coupling, if the model is specified as ‘non-linear’, then each matrix will include all frequency couplings, including within and cross-frequency couplings. The ‘A’ matrix describes the coupling strength between the source and target frequencies, dependent on exogenous inputs, for all trials (i.e. the Go and NoGo accurate trials). The ‘B’ matrix describes the coupling strength between the source and target frequencies, dependent on the experimental manipulation (i.e. the NoGo versus the Go trials). The last step in the DCM is to identify the optimal model that best supports the observed data using Bayesian model selection based on free-energy estimates of the log model evidence.

In this study, the model architecture included three predefined regions based on the well-described response inhibition network: the left motor cortex, preSMA, and right IFG (Montreal Neurological Institute, MNI coordinates: −37 −25 64; −4 4 60; 48 18 −2). The coordinates for preSMA and motor cortex were based on a functional MRI meta-analysis of right hand motor control (Mayka *et al.*, 2006). The right IFG coordinate was based on a recent NoGo inhibition study (Ye *et al.*, 2014).

The set of models designed included two families defined by the possibility for linear only or combined linear and non-linear coupling. Within each family, seven model architectures were examined to test alternate hypotheses about the contribution of power couplings between regions. All models assumed reciprocal coupling between the three regions for the exogenous task-related perturbations. For the condition-dependent perturbations the model space was varied, allowing either: all reciprocal connections, two sets of connections, or just one set of connections to be modulated by condition. All self-connections were modulated in all models. The cortical driving input was applied to the sources in the IFG and preSMA (Rae *et al.*, 2015).

The DCM included only the accurate Go and NoGo trials and the gradiometer channels for the dipole model fit. The time-frequency decomposition used similar processes to the sensor space analysis: Morlet wavelets with a factor of 5 were computed across a frequency bandwidth 4–80 Hz. The data were reduced to four frequency modes obtained from a singular value decomposition of the spectral power. In our dataset these four modes explained 96% of the variance in both groups [standard deviation (SD): Controls = 2.3%; bvFTD = 9.7%]. The models predicted frequency dynamics across a window of −50 to 1200 ms to include the ERD and ERS. Onset priors, reflecting stimulus input, were set to the default 60 ms post-stimulus presentation with a standard deviation of 16 ms, adjusted during the process of model fitting with default variance. The conditional modulation of the model (as represented by the ‘B’ matrix) compared accurate NoGo versus accurate Go trials.

Bayesian model selection was used to identify the model with the best fit to the data using free-energy estimates of the log model evidence. A two-step procedure was used. First, to identify whether cross-frequency coupling was an important feature, the seven linear and seven non-linear models were compared using a family-based model comparison procedure (Penny *et al.*, 2010). Second, the seven individual models within the winning family were compared to identify the best model architecture. The subject-specific frequency-frequency parameter estimations of the winning model were entered into two flexible factorial ANOVAs, one for the connection parameters of all trials (A matrix) and one for the parameters of NoGo versus Go trials (B matrix). The effects of interest were the interactions between group and connection (two groups × nine connections), and also the difference between controls and patients.

#### MRI

A T_1_-weighted structural image (magnetization prepared rapid acquisition gradient echo, MPRAGE) was obtained from each subject (repetition time 2250 ms, echo time 2.99 ms, flip angle 9°, inversion time 900 ms, 256 × 256 × 192 isotropic 1 mm voxels) to co-register the MEG data and to enable subject-specific modelling of the lead field for the DCM analyses. These images were also included in the voxel-based morphometry (VBM).

The VBM analysis used SPM 12 (www.fil.ion.ucl.ac.uk/spm) and the DARTEL toolbox (Ashburner, 2007), and followed the steps suggested by Ashburner (2007). The T_1_ images for each participant were segmented into grey, white, and CSF tissue classes and together used to create a study-specific group template to improve intersubject alignment during normalization. The template was registered to MNI space and used to generate Jacobian scaled modulated grey and white matter images from each subject that were spatially normalized to MNI space and smoothed with an 8-mm full-width at half-maximum (kernel.

Two general linear models were used to examine the differences in the grey and white matter images between patients and controls. For each general linear model, total intracranial volumes from each subject were included as nuisance covariates to correct for intersubject differences in global brain volume. Age was also included as a covariate. Statistical maps were thresholded with a cluster-based familywise error correction *P* < 0.05 (after *P* < 0.001 voxelwise uncorrected threshold). A Bayesian estimation of the same contrast was performed and then subjected to a null hypothesis test, providing a statistical map of the posterior probability at each voxel. Voxels considered significant exceeded 95% confidence threshold and had a volume threshold greater than 0.7%. These voxels represented regions that had strong evidence for normal cortical volume.

### Data availability

The dataset generated and analysed during the current study is available from the corresponding author on request from qualified researchers for non-commercial research purposes. A material transfer agreement may be required.

## Results

### Participants and behaviour

Table 1 presents demographic data and results from cognitive tests. Among the 18 patients, seven were taking either trazadone, citalopram or fluoxetine, and one was taking low dose chlorpromazine. No patients were taking cholinesterase inhibitors, benzodiazepines or other GABA agonists. Compliance with task demands was high following practice trials, with both groups performing sufficiently well on the task to interpret the MEG: mean Go accuracy: Controls 99.2%, bvFTD 92.7%; mean NoGo accuracy: Controls 93.8%, bvFTD 90.5%. The bvFTD group made significantly more omission errors on Go trials [W(38) = 298, *P* < 0.05, Cohen’s *d* = 0.65], but at a group level they did not make significantly more commission errors on NoGo trials [W(30) = 176, *P* > 0.05, Cohen’s *d* − 0.02]. Mean reaction times: Controls Go trials = 290 ms, NoGo trials = 223 ms, *t*(18) = 5.7, *P* < 0.05; bvFTD Go trials 488 ms, NoGo trials = 445 ms, *t*(13) = 2, *P* > 0.05. Compared to controls, the bvFTD group were significantly slower when responding to the Go trials [mean reaction times: Controls Go = 290 ms, bvFTD Go 488 ms; *t*(36) = 5, *P* < 0.001, Cohen’s *d* = − 1.6] and they were slower when making commission errors on the NoGo trials [mean reaction times: Controls NoGo = 223 ms, bvFTD NoGo = 445 ms; *t*(31) = 3.4, *P* < 0.05, Cohen’s *d* = − 1.2]. A Bayes factor analysis of the Go reaction time distributions provided very strong evidence (Bayes factor = 1248) for a difference between groups. For group differences in NoGo reaction time and Go accuracy, there was strong evidence (Bayes factor = 21) for a difference, but NoGo accuracy, there was no evidence for a group difference (Bayes factor = 0.3).

### Time-frequency analysis

**Figure 2:**
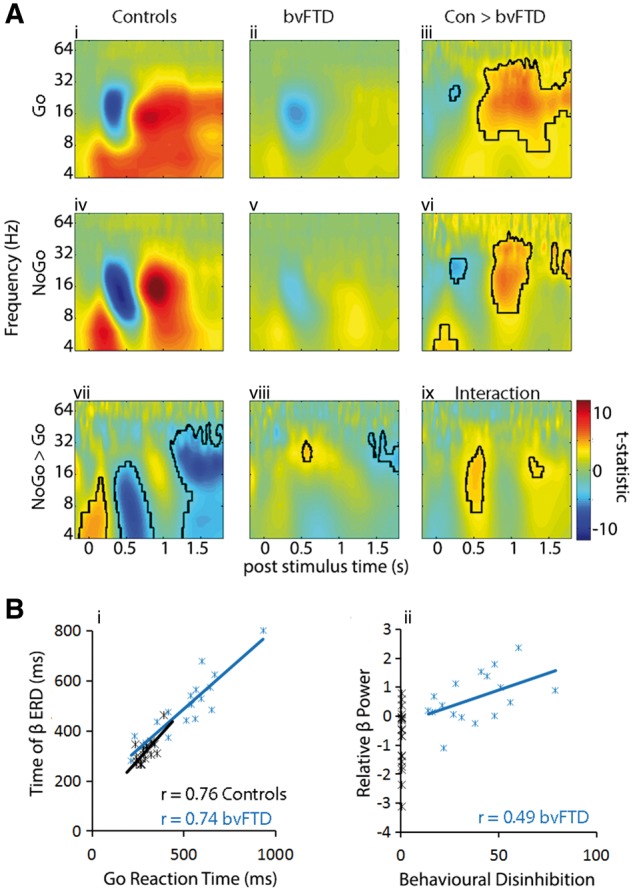
**Time frequency spectra and relationship with behaviour.** (**A**) Time frequency spectra for controls and bvFTD patients for successful Go and NoGo trials. A clear ERD/ERS in the beta/alpha bands and an early increase in theta are evident, which are diminished in patients. Contrasts between conditions (**vi–vii**) and between groups (**iii**, **vi**) are plotted with significant statistical thresholds outlined in black (F tests, *P* < 0.05 clusterwise corrected after *P* < 0.001 voxelwise threshold). The interaction (Go versus NoGo × Patients versus Controls, **ix**) reveals two windows of significance, used for further analyses. [**B**(**i**)] Time of peak beta desynchronization plotted against reaction times, revealing a tight link between desynchronization and movement. [**B**(**ii**)] Plot of relative beta power (the difference between Go and NoGo trials), at the peak of the significant interaction (18 Hz at 540 ms), which correlates with behavioural measures of disinhibition from the CBI. The correlation is positive, indicating that patients who are more behaviourally disinhibited have less desynchronization during successful NoGo trials.

The time-frequency spectra for the successful Go and NoGo conditions (Fig. 2A) confirms the ERD in power over 300–600 ms in the alpha and beta bands (8–30 Hz) and an ERS in this bandwidth after 600 ms. The observed spectra for the patient group followed a similar but markedly attenuated pattern. To investigate the relationship between the ERD and response time, the time of peak beta ERD for successful Go trials was correlated with mean reaction time (Fig. 2B). This was significant in both groups (Controls: Pearson’s r = 0.71, *P* < 0.05; bvFTD patients Pearson’s r = 0.91, *P* < 0.05), confirming that ERD is an important factor in the timing of the button press response.

An ANOVA contrasting the NoGo and Go conditions for controls revealed a significant increase in theta and a greater ERD for the NoGo condition, while the Go trials had a greater and prolonged ERS. In patients, the differences between conditions were small, with reduced beta ERD for the NoGo compared to the Go condition, and a late rebound in the Go trials. A significant interaction was present between the two groups and conditions [peak of cluster: 18 Hz at 540 ms, and also 18 Hz at 1322 ms, Fig. 2A(ix)], with a greater ERD for Go trials compared to NoGo trials in patients and the opposite pattern for controls. This interaction indicates that in controls, successful inhibition occurs despite desynchronization of low frequency bands, but in patients, successful inhibition was characterized by minimal low frequency power changes. In patients, the difference between Go and NoGo trials at the peak of the interaction significantly correlated with behavioural disinhibition (composite score from the CBI measures of disinhibition) (Borroni *et al.*, 2012; Hughes *et al.*, 2015) (Pearson’s r = 0.49, *P* < 0.05, Fig. 2B). This suggests that in those patients who were more behaviourally disinhibited, successful NoGo trials were characterized by less desynchronization in beta power compared to the Go trials.

### Network dynamics

**Figure 3:**
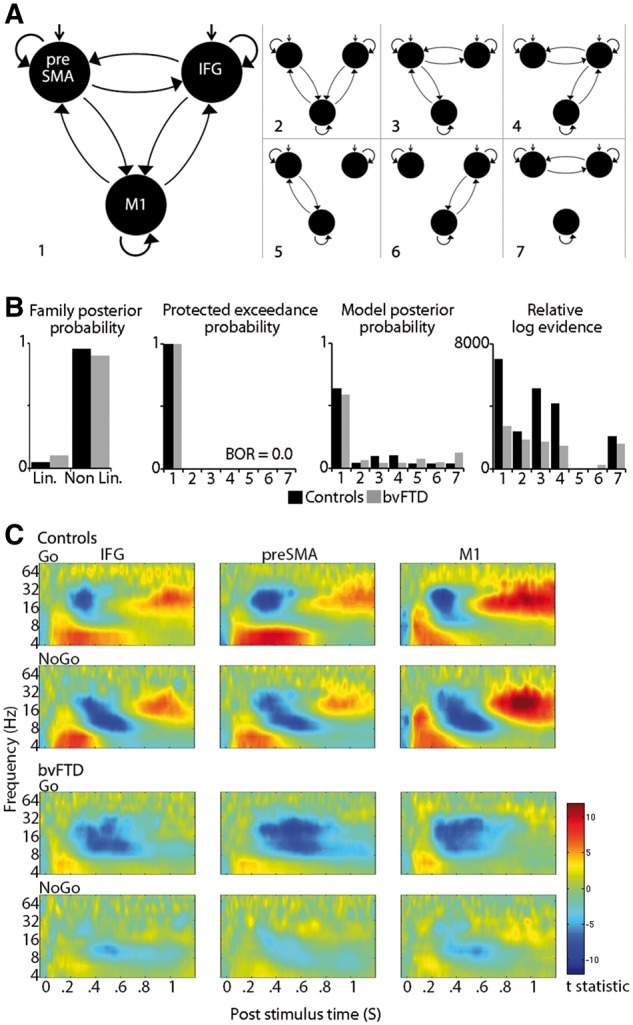
**Details of dynamic causal models.** (**A**) DCM model space with seven different architectures. (**B**) Bayesian model selection reveals the non-linear family best fitted the data, which allows for cross-frequency coupling. Of the seven models within the non-linear family, Model 1 was the winning model, with task modulation of reciprocal connections between all regions. For both controls and bvFTD patients, Model 1 had the greatest exceedance and posterior probabilities and a difference in log evidence between the winning and second best model > 3. (**C**) Time-frequency spectra for each region, for each condition and group.

Fourteen generative models were inverted and compared: seven with linear frequency dynamics and seven with both linear and non-linear dynamics that allow for cross-frequency coupling. Each set of seven models included the same three nodes (Fig. 3A). Bayesian model family comparisons (Fig. 3B) identified the non-linear family as the most likely, given the data, for both groups (family posterior probability: Controls = 0.95, bvFTD = 0.89). Within this family of non-linear models, model evidence strongly favoured Model 1 (Fig. 3B), the model with task modulations of the reciprocal connections between all three regions. This model was identified as the most likely for both groups. Exceedance probability: controls = 0.99, patients = 0.99; posterior probability: controls = 0.64, patients = 0.58; relative log model evidence: controls = 7027, patients = 2767, both these model evidence values exceeded the second most likely model for each group by more than 5, equivalent to a Bayes factor of 150, which is considered as very strong evidence for that model (Raftery, 1995).

The time-frequency spectra for the three sources used in the DCM (Fig. 3C) follow the time-frequency pattern of the averaged sensor space plots, i.e. an early increase in theta, an alpha-beta desynchronization succeeded by a rebound. A time locked gamma increase is also observed. This pattern is diminished in patients with bvFTD.

**Figure 4:**
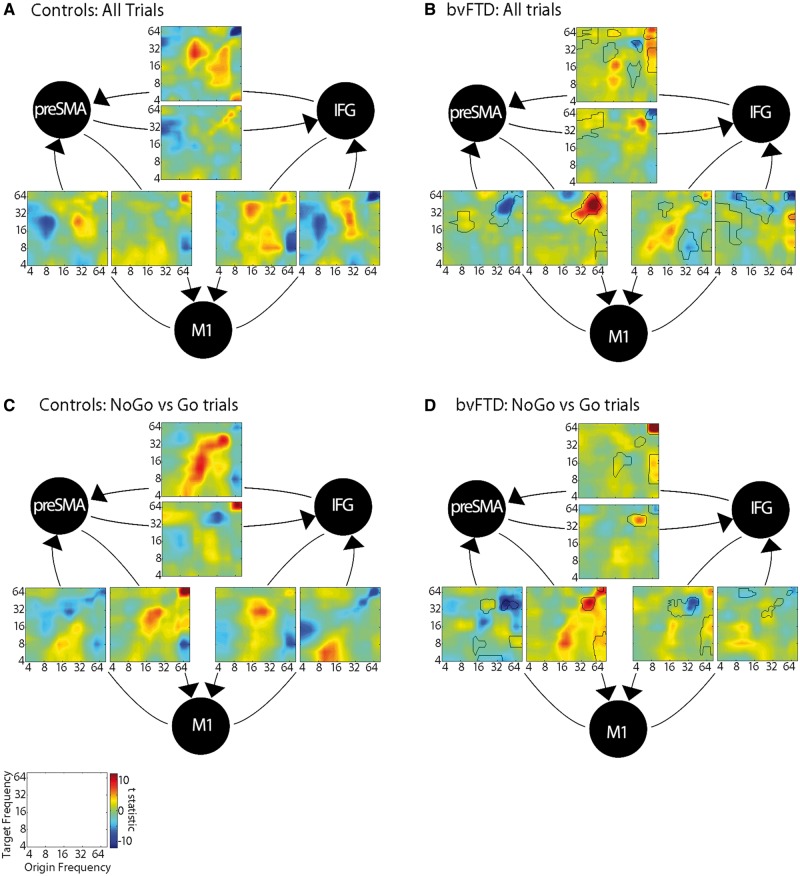
**Statistical parametric maps of the frequency to frequency estimations for each interregional connection of the winning model, plotted on a log scale in Hz.** Negative (blue) values represent a suppression effect: a power increase in the origin frequency decreases power in the target region, and positive (red) values represent an increase in origin frequency that increases power in the target region. In **B** and **D** the differences between the bvFTD and control groups are outlined in black (*P* < 0.05 FWE cluster-wise corrected after *P* < 0.001 voxel-wise correction).

The frequency-frequency plots (Fig. 4) reveal the cross-frequency couplings. In controls (Fig. 4A), beta suppression (12–30 Hz) is represented by negative cross-frequency coupling with theta-alpha (4–12 Hz), and high gamma (60–80 Hz) bands. Specifically, M1 theta-alpha coupling with beta in preSMA and IFG, and these regions drive a gamma to beta coupling with M1. The beta rebound is driven by an increase in within-frequency beta to beta coupling between all regions. In patients with bvFTD (Fig. 4B), the within and cross-frequency coupling between regions is significantly diminished. However, an increase in reciprocal gamma to gamma (60 to 60 Hz) coupling is evident between M1 and preSMA.

**Figure 5:**
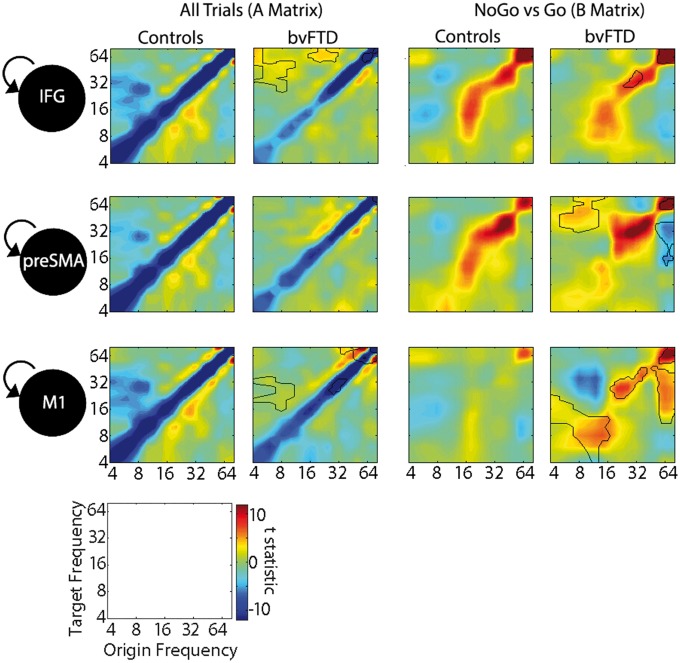
**Statistical parametric maps of the frequency to frequency estimations for each of the self-connections of the winning model.** Negative (blue) values represent a suppression effect: a power increase in the origin frequency decreases power in the target region, and positive (red) values represent an increase in origin frequency that increases power in the target region. The significant cluster differences between the bvFTD and control groups are outlined in black (*P* < 0.05 FWE cluster-wise corrected after *P* < 0.001 voxel-wise correction). The self-connections for all trials (A matrix) show within-frequency couplings that are strongly negative on the diagonal. This feature of cortical networks is included in dynamic causal models, in which estimations of intrinsic connections depend on self-inhibition for stability, and this constraint establishes a negative prior on the coupling parameter (Friston *et al.*, 2003).

The B matrix represents the difference between the NoGo and Go conditions, identifying couplings contributing to response inhibition. For controls (Fig. 4C) the difference in beta desynchronization between the conditions is relatively unchanged, but the rebound is enhanced. In M1 there is greater within-frequency beta and gamma coupling from IFG and preSMA, and from IFG to preSMA. There is also enhanced theta in IFG and preSMA from beta couplings with M1. These enhanced connections indicate how a button press is prevented in the NoGo trials. In patients (Fig. 4D), the reciprocal frequency couplings are significantly reduced, and particularly notable is the increase in positive and negative gamma to gamma coupling between preSMA and M1, and a distinct loss of cross-frequency coupling from IFG to preSMA and M1. The self-connections (Fig. 5) also reveal a beta desynchronization by theta and alpha to beta couplings, which are diminished in patients. The shift in connectivity from IFG, onto preSMA–M1 circuits during successful Go and NoGo trials has mechanistic implications for behavioural control.

### Voxel-based morphometry

The VBM confirmed extensive grey and white matter atrophy for the patient group, particularly in the prefrontal and temporal cortex (Fig. 1A). The null hypothesis Bayesian estimation indicated posterior regions as having normal cortical volume. Of note, the motor and presupplementary motor cortex were indicated to have normal cortical volume, while the right inferior frontal gyrus was atrophic. However, it is important to note that atrophy as measured using VBM is a late rather than early correlate of neuropathology and is indicative of further degeneration. Features such as tau-positive inclusions and loss of synaptic density may be present in the regions with normal cortical volume. The table of peak atrophy and cross-sectional views of coronal, axial, sagittal planes are available in the Supplementary material.

## Discussion

The principal results of this study are the changes in cortical oscillatory dynamics and frontal connectivity during response inhibition in patients with frontotemporal dementia. Behavioural variant FTD attenuated the normal pattern of event-related beta desynchronization and rebound, in proportion to carer observations of everyday challenging and disinhibited behaviours. The reduction in beta resynchronization and re-synchronization was further associated with reorganization of interregional connectivity: including the loss of cross-frequency coupling in connections of the inferior frontal gyrus, and the enhancement of gamma coupling between the preSMA and motor cortex.

We propose that the loss of beta desynchronization is central to understanding the link between pathophysiology and behaviour in frontotemporal dementia. Beta desynchronization (ERD) has been considered an index of movement planning, preparation and execution (Pfurtscheller and Lopes da Silva, 1999; Pfurtscheller *et al.*, 2013) but can also be observed outside the core motor system and may reflect a generalized signal for cognitive or behavioural state transitions, encompassing set changes as well as motor acts (Engel and Fries, 2010). Changes in alpha and beta oscillations are also markers of social cognition (Billeke *et al.*, 2013, 2014), which are reduced in frontal regions in patients with bvFTD (Melloni *et al.*, 2016; Ibanez *et al.*, 2017). This converging evidence for loss of oscillatory power with disease suggests a common mechanism for behavioural disinhibition, within a broader model of contextually inappropriate actions and disinhibited behaviours.

In our paradigm, despite the diminished ERD, patients’ accuracy on Go trials was maintained and we observed a strong relationship between the latency of the peak desynchronization and reaction time. This is consistent with a lower physiological threshold for movement in FTD, and may lead to inappropriate or ill-considered actions. Correspondingly, the ERD during successful NoGo inhibition trials was also diminished in patients, and importantly this correlated with clinical measures of disinhibition: higher levels of behavioural disinhibition were related to a more attenuated desynchronization. In other words, successful inhibition in the more disinhibited patients continues to depend upon a degree of maintenance of beta power. In contrast, the ERD was present in the control group during successful NoGo trials, which may represent preparatory or initiated actions induced by the regular repetitive Go trials (Swann *et al.*, 2009; Pfurtscheller *et al.*, 2013).

The differences between patients and controls within distinct frequency bands indicates a divergence in the mechanisms for successful motor control in health and dementia. The mechanism of this divergence is revealed by the most likely of the generative dynamic causal models. The frequency-specific connectivity between three key cortical regions revealed altered cross-frequency coupling in patients, leading to the diminished beta desynchronization. This can be interpreted in light of the established pattern of preferential burden of pathology in the superficial cortical layers (Kersaitis *et al.*, 2004; Irwin *et al.*, 2016), together with the known physiological properties of inter-laminar connectivity (Buffalo *et al.*, 2011).

In the control group, the ERD during the Go and NoGo trials was associated with asymmetric reciprocal connectivity between IFG, preSMA and motor cortex: gamma oscillations in IFG and preSMA elicited a beta desynchronization in primary motor cortex, while increased theta and alpha power in the motor cortex initiated the beta ERD in the IFG and preSMA. We suggest that these reciprocal couplings represent common movement preparation mechanisms for both trials. The beta rebound was characterized by beta-to-beta couplings in both trials, but differences in the direction of couplings indicate the mechanisms of inhibition: NoGo trials were characterized by enhanced positive within-frequency couplings in the beta and gamma frequencies from IFG and preSMA to motor cortex, and a loss of beta-to-beta coupling from motor cortex to IFG and preSMA, suggesting a predominantly top-down influence on motor cortex to inhibit responses.

Converging evidence from EEG, TMS and ECG, supports a model of hierarchical frequency-specific interactions between prefrontal, premotor and motor cortex regulating motor responses. As we have shown here, response inhibition has been associated with enhanced beta and gamma oscillatory coupling between right inferior prefrontal gyrus and preSMA with left primary motor cortex (Swann *et al.*, 2009, 2012; Picazio *et al.*, 2014). These results are leading towards a consensus for beta oscillations as an index of inhibition, although the temporal precision of couplings between regions is still to be resolved (Picazio *et al.*, 2014). Other task demands may also influence the timing and frequency of oscillatory changes. For example, during stop trials, gamma increases in preSMA precede right IFG (Swann *et al.*, 2012), but might be related to the salience of the cue rather than inhibition (Fonken *et al.*, 2016).

In patients with bvFTD, the cross-frequency couplings were minimal, particularly in connections with the IFG. The loss of these couplings can be interpreted in the context of the known distribution of pathology, topography of neural connectivity and the biophysics of different rhythms.

In bvFTD, the burden of pathology is greatest in superficial layers of the frontal cortex layers (Kersaitis *et al.*, 2004; Irwin *et al.*, 2016) with widespread white matter pathology (Irwin *et al.*, 2016). In health, neuronal firing patterns of this superficial layer have specific physiological and dynamical properties: loops of feedback inhibition between fast spiking GABAergic interneurons and pyramidal cells generate gamma oscillations (30–80 Hz) (Kopell *et al.*, 2010), and drive connections to target cells in granular and infragranular cortical layers (Jensen *et al.*, 2015). In bvFTD, the reduction of ‘prefrontal gamma’ to ‘motor beta’ coupling may represent the loss of these connections, which limits the beta ERD. The mechanisms sustaining theta to beta oscillations are neurochemically less well specified, but these frequencies are considered to represent primarily feedback information, because deeper cortical layers oscillate at lower frequencies, and these layers innervate superficial cortical layers (Buffalo *et al.*, 2011; Barbas, 2015). Furthermore, the slower firing properties of deeper layers are more appropriate to modulatory feedback, synchronizing cell assemblies over longer conduction delays (Kopell *et al.*, 2000). In FTD, the loss of these forward and backward connections between the IFG, preSMA and motor cortex would disrupt the desynchronization and resynchronization of the beta band underlying movement control. The method of DCM used in this study does not enable us to examine the dynamics of different cortical laminae directly; however, generative models used for DCM can in principle encompass a canonical microcircuit with differentiation of superficial and deep pyramidal cell populations, and future analyses using such models may provide further insights into the hypothesis of laminar specificity (Bhatt *et al.*, 2016; Rosch *et al.*, 2017).

Despite the loss of cross-frequency couplings and a diminished ERD, patients do respond well to Go trials and we speculate whether patients’ physiological state is one of a relative readiness to move. Such claims have been formally tested in patients with frontotemporal lobar degeneration, for example in an oculomotor Go-NoGo task, patients with progressive supranuclear palsy manifest a bias to respond in Go trials despite akinesia (Zhang *et al.*, 2016). The ‘readiness to move’ may explain the disinhibited nature of the patients, supported by the evidence that even when successfully moving or inhibiting a movement, desynchronization is limited.

During NoGo response inhibition, the positive beta couplings between regions were also diminished in bvFTD, but the beta power was enhanced by self-couplings within each region, particularly in the motor cortex. Moreover, an increase in gamma-to-gamma coupling between the preSMA and M1 was observed in the patient group that was not present in controls. This gamma hyper-synchronization between regions that are less severely affected by the pathology of bvFTD has been noted previously, in an auditory paradigm (Hughes and Rowe, 2013). Together with increased local beta synchrony, it may represent a shift in connectivity away from long-range interlaminar prefrontal connectivity towards local circuits. We suggest that this shift is in response to pathological disruption, and for patients who were able inhibit some responses to NoGo cues, it may facilitate task performance.

There are limitations to this study. Whilst we explicitly examined the modelled interactions between three frontal cortical regions and suggest explanations of beta desynchronization based on cortical network dynamics, we acknowledge that additional regions have been associated with response inhibition (Ye *et al.*, 2014; Rae *et al.*, 2016) but deeper sources are more difficult to detect with MEG, as the signal attenuates rapidly with distance from the sensors, yet may still contribute to the scalp signal (Attal *et al.*, 2007). Within a broader model of disinhibition in patients, additional prefrontal regions may also be relevant (Ibanez and Manes, 2012; O’Callaghan *et al.*, 2013; Ibanez *et al.*, 2017). We have used a specific task, based on the normative inhibitory control model, as a proxy to understand behaviour. The relationship between oscillatory power with everyday behaviour (as measured by the CBI) contributes to the validation of our approach. However, other tasks and modelling approaches would be required to directly link motor control circuits to the social and emotional regulation, analogous to the DCM modelling of memory and emotion interaction. In addition, our network modelling of the 1200 ms window reveals changes in the frequency dynamics across this window, the temporal specificity of couplings are not determined: the changes in frequency couplings are interpreted in the context of the time-frequency data as recommended (van Wijk *et al.*, 2013).

The heterogeneity of the bvFTD group must also be considered, particularly in terms of (i) variable reaction times during the Go trials, although our analyses compared Go with NoGo trials to ameliorate the effects of variability in responding; (ii) variable pathology, with a likely mixture of tau and TDP43 pathologies in the group; and (iii) concurrent drug treatment that may differentially affect neuronal responses. We cannot wholly rule out an effect of medication, especially chronic serotonergic medication in 7 of 18 patients, which may influence frontal cortical responses during inhibition (Hughes *et al.*, 2015). However, changes in cortical oscillations are not typically associated with SSRI treatment, but instead may be associated with GABA agonists (Muthukumaraswamy *et al.*, 2013), noting that none of the patients in this study were taking primary GABAergic medication.

In conclusion, we suggest that the IFG, preSMA and motor cortex form a functionally and structurally connected network, which mediates optimal motor performance by frequency-specific directional driving and modulating oscillations. We propose that disinhibition in bvFTD results from the loss of cross-frequency connectivity between these regions. The increase in high-frequency coupling from preSMA to motor cortex may be compensatory, or may reflect the loss of prefrontal regulation of motor cortex (Sharma *et al.*, 2009) and other non-prefrontal regions, such as the basal ganglia, which are implicated in response inhibition (Rae *et al.*, 2016). The result is impaired beta desychronization, especially when response inhibition is required, with slowing of reaction times and an increase in day to day disinhibition as observed by carers. We suggest that lamina-specific cell loss, and GABAergic loss caused by FTD (Murley and Rowe, 2018) contribute to the behaviourally relevant neurophysiological patterns, exacerbating contextually inappropriate behaviours. This provides a potential pharmacological target and a precise, but clinically relevant, neurophysiological signature for future experimental medicines studies in FTD.

## Funding

This work was primarily funded by the Wellcome Trust (103838) with additional support from the Medical Research Council (MC-A060-5PQ30, and RG62761) and the NIHR Cambridge Biomedical Research Centre and Cambridge Brain Bank. The BCNI is supported by a joint award from the Wellcome Trust and Medical Research Council.

## Supplementary material

Supplementary material is available at *Brain* online.

## Supplementary Material

Supplementary Data

## Abbreviations

bvFTD: behavioural variant frontotemporal dementia
ERD/S: event-related desynchronization/synchronization
IFG: inferior frontal gyrus
preSMA: pre-supplementary motor area
