# Supplementary material for: Reorganization of cortical oscillatory dynamics underlying disinhibition in frontotemporal dementia
Source: Brain. 2018 Jul 9;141(8):2486–99. doi: 10.1093/brain/awy176 (PMC6061789; doi:10.1093/brain/awy176)
Supplement: Supplementary Data [file awy176_supp.zip › awy176-suppl_data/brain-2018-00004-File007.pdf]

Supplementary

Figure 1

Figure 1 Supplementary. Voxel-based morphometry analyses of atrophic grey (red) and white matter (blue) in patients with bvFTD compared to the control group. Significant clusters are overlaid on MNI image, in coronal, axial and sagittal views. Clusters are FWE corrected (p<0.05) after a voxel wise correction (p<0.001).

Table 1: Regions of peaks in clusters of grey matter atrophy

**Region**	**Cluster**	**x**	**y**	**z**	**peak F**	**Peak z**
Left middle temporal gyrus	92747	51	-22	-16	62.16	5.68
Left insula / inferior frontal gyrus		-26	21	-8	55.09	5.46
Right temporal pole		44	4	-28	50.69	5.32
Left supramarginal gyrus	1891	-50	-32	45	55.55	5.48
Left superior parietal lobe		-28	-40	45	17.67	3.53
Right superior parietal lobe	1390	18	-54	51	30.35	4.42
Right angular gyrus		27	-54	42	19.9	3.72
Right precuneus		15	-56	34	18.95	3.64
