# Supplementary figures and images for: Reorganization of cortical oscillatory dynamics underlying disinhibition in frontotemporal dementia
Source: Brain. 2018 Jul 9;141(8):2486–99. doi: 10.1093/brain/awy176 (PMC6061789; doi:10.1093/brain/awy176)

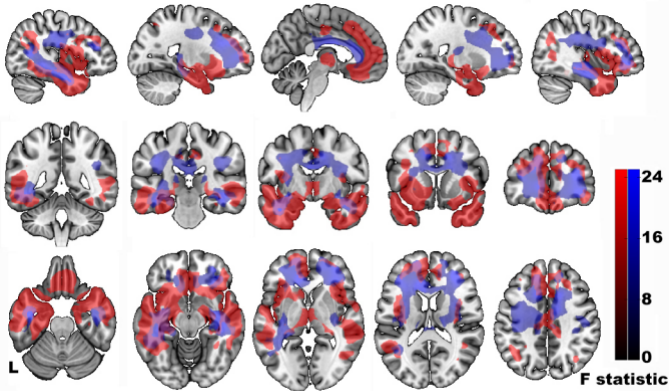

Supplement: Supplementary Data [file awy176_supp.zip › awy176-suppl_data/brain-2018-00004-File008.pdf]
